# Case Report: Management of Delusional Parasitosis With Parkinsonism

**DOI:** 10.1192/bjo.2026.11900

**Published:** 2026-06-30

**Authors:** Deoman Gurung, Olivia Udeagu, Sururat Ibrahim

**Affiliations:** lancashire care and south Cumbria NHS trust, blackburn, United Kingdom

## Abstract

**Aims:**

The co-occurrence of delusional parasitosis (primary Ekbom syndrome) with idiopathic Parkinson’s disease is considered rare. Most literature consists of individual case reports or small series, indicating it is an uncommon but recognised clinical phenomenon. The rarity is compounded by the therapeutic challenge it presents.

**Methods:**

A 74-year-old gentleman of Caribbean origin, with a longstanding diagnosis of persistent delusional disorder (delusional parasitosis, F22.0), was admitted under Section 3 of the Mental Health Act. He had a history of non-concordance with olanzapine for three years, resulting in significant clinical deterioration. Comorbidities included idiopathic Parkinson’s disease (also non-concordant with medication) and prostate cancer in remission.

On presentation, he exhibited classic Ekbom syndrome: believing bugs infested his body, he applied yoghurt to his skin and stored debris. Profound self-neglect, hoarding of soiled items and out-of-date food, and environmental squalor were noted. Significant weight loss, worsening mobility, and cognitive decline were reported.

**Results:**

Management was complicated by the contraindication of many antipsychotics in Parkinsonism, where they exacerbate motor symptoms. Olanzapine was briefly restarted but poorly tolerated. Aripiprazole was initiated cautiously at a low dose (max 25mg daily) with monitoring for akathisia. Procyclidine effectively managed resultant hypersalivation. His Parkinson’s medications were recommenced.

Pharmacological guidance, per Maudsley’s guidelines, restricts options. Risperidone and typical antipsychotics like flupentixol are contraindicated due to high extra-pyramidal side effect risk. While quetiapine has marginal efficacy, it is often trialled before clozapine. In this case, aripiprazole was selected as a potentially safer atypical agent.

**Conclusion:**

This case illustrates the complex interplay between psychotic illness and neurodegenerative disease, highlighting the severely limited and high-risk antipsychotic options available. Multidisciplinary management, including SALT input (showing dietary improvement), remains essential for this vulnerable patient with concurrent severe mental illness, physical comorbidity, and challenging medication non-adherence.

